# A Mobile App With Optical Imaging for the Self-Management of Hand Rheumatoid Arthritis: Pilot Study

**DOI:** 10.2196/12221

**Published:** 2018-10-29

**Authors:** Elizabeth Mollard, Kaleb Michaud

**Affiliations:** 1 College of Nursing Lincoln Division University of Nebraska Medical Center Lincoln, NE United States; 2 Division of Rheumatology and Immunology University of Nebraska Medical Center Omaha, NE United States; 3 FORWARD, The National Databank for Rheumatic Diseases Wichita, KS United States

**Keywords:** optical imaging, mobile applications, self-management, rheumatoid arthritis, self efficacy, patient activation, pilot study

## Abstract

**Background:**

Patient outcomes are improved and the burden to the health care system is reduced when individuals are active self-managers of their own health. There is a need for technology that facilitates self-management of rheumatoid arthritis (RA) and can reduce the number of patient visits, promptly identify treatment needs, and reduce the costs associated with poor RA management. A mobile app named LiveWith Arthritis (eTreatMD, Vancouver, BC) has been developed that allows patients with RA to use their mobile device to regularly collect self-management data and to take objective measurements of the impact of RA on their finger joints using optical imaging technology.

**Objective:**

The objectives of this pilot study were to (1) gather preliminary data as to whether a mobile app with hand optical imaging capabilities improves self-management behaviors (self-efficacy in managing symptoms and patient activation), (2) determine if app use shows promise in improving health outcomes (Pain, Health Assessment Questionnaire-II [HAQ-II]), and (3) determine barriers to using the mobile app in adults with RA.

**Methods:**

This pilot study used a mixed-methods design. The quantitative portion was a traditional 2-group experimental design, and the qualitative portion was a follow-up telephone interview for intervention participants who did not complete the study. Measures of self-management included the Patient-Reported Outcomes Measurement Information System (PROMIS) self-efficacy in managing symptoms (P-SEMS) and Patient Activation Measure (PAM). Health outcomes included pain by Visual Analog Scale and disability by HAQ-II.

**Results:**

The final sample consisted of 21 intervention participants and 15 controls. There was a statistically significant improvement in P-SEMS and promising trends for improvement in PAM, HAQ-II, and pain scores for participants who used the app. Of the intervention participants who did not complete the study, 12 completed the qualitative interview on barriers to use. Qualitative content analysis revealed 3 themes for barriers to using the app, including (1) frustration with technology, (2) RA made the app difficult to use, and (3) satisfaction with current self-management system.

**Conclusions:**

The LiveWith Arthritis app shows promise for improving self-management behaviors and health outcomes in adults with RA. Future study with a larger sample size is required to confirm findings. Initial app experience is important for adoption and continual use of the app. Individuals with significant disability to the hand would benefit from voice-activated app features. Participants who already have a system of managing their RA may not feel compelled to switch methods, even when a novel optical imaging feature is available.

## Introduction

### Background

Rheumatoid arthritis (RA) is a chronic and debilitating systemic inflammatory disease that destroys joints throughout the body. Although RA can manifest in a variety of joints, most patients experience RA symptoms in their hands and wrists [1]. Hand deformities are a common feature of RA as are their related physical deficits such as reduced grip strength and pain which decrease the ability of the individual to perform activities of daily living [2]. Each episode of RA-induced inflammation in the synovial joints of the hand is a painful predictor of future bone loss and long-term deformity [3]. RA induced damage and deformity can be prevented through reduction of individual patient triggers that induce inflammatory *flares* and by early and consistent use of disease-modifying antirheumatic drugs and other pharmacotherapeutics [4].

Although the hand is the primary area for painful and disabling symptoms, it is also an area that can be monitored to predict disease flares and monitor disease progression. While clinicians can use radiographic imaging to monitor RA progression, measurement is infrequent due to the cost of implementation, the availability of radiologists for interpretation, and radiation exposure risks. The use of noninvasive optical imaging methods to analyze interphalangeal joint disease is an emerging technology used mostly in clinical settings [5]. Optical imaging includes optical coherence tomography, diffuse optical tomography, laser transillumination imaging, photoacoustic tomography, and digital imaging of the hands [6-8]. These techniques have historically required specialized equipment for measurement and interpretation. However, with the advances in camera and software technologies in smartphones and tablets, these optical imaging capabilities are now available in consumer devices [9].

In addition to monitoring, self-management of the day-to-day symptoms of RA is critical to reducing disease progression. The Institute of Medicine states that self-management is 1 of the 20 most urgent areas of need to improve health care quality in the United States [10]. Self-management is a daily, interactive process that engages the individual, encourages them to monitor their illness, and empowers them to make decisions and develop and use strategies to maintain an improved quality of life. Active patient participation in the management of an individual’s health and disease has been shown to improve health outcomes, including reduction of pain and disability while providing a greater sense of well-being [11]. Increased self-management behaviors are associated with reduced health care utilization and related costs [12]. Key predictors of self-management behaviors are self-efficacy in managing symptoms and patient activation. An individual’s perceived self-efficacy refers to their confidence in their ability to be successful and to complete tasks to manage their symptoms and is associated with reduced disability and pain [13-15]. Patient activation is an important element of self-management that includes having the skills and confidence to become actively engaged in one’s own health care [16].

### Objectives

The objectives of this pilot study were to (1) gather preliminary data as to whether a mobile app with hand optical imaging capabilities improves self-management behaviors (self-efficacy in managing symptoms and patient activation), (2) determine if app use shows promise in improving health outcomes (Pain, Health Assessment Questionnaire-II [HAQ-II]), and (3) determine barriers to use of the mobile app in adults with RA.

## Methods

### Materials

**Figure 1:**
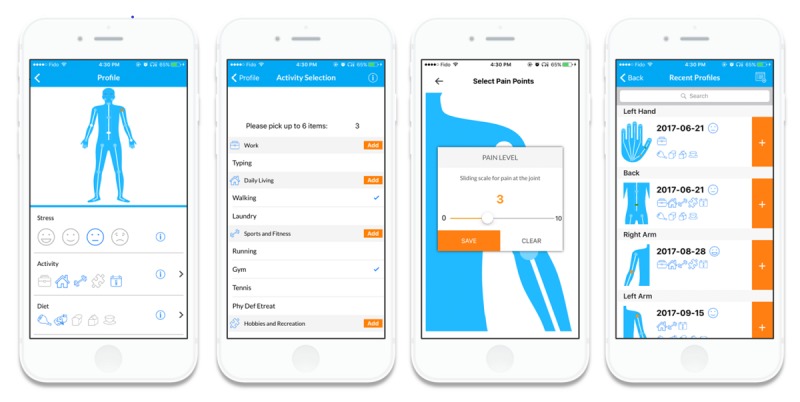
App self-management features.

The LiveWith Arthritis app is a mobile app that allows a patient to monitor the progression of RA inflammation and deformity in their hands using optical imaging from a mobile device camera. The app also supports self-management behaviors with features to monitor and manage the variables associated with RA such as pain levels, treatments, and other lifestyle and environmental data (eg, diet, activity, and weather; see Figure 1). The system can provide reports for individuals, clinicians, and caregivers that might help identify aspects of patient lifestyle that make their arthritis better or worse and let them compare the effectiveness of different treatments. This app is intended to engage the patient in their treatment, facilitate discussions between patient and clinician, and provide information on which clinical decisions can be based. Using this app may help with the day-to-day reduction of RA symptoms and build confidence in the patient in the management of their condition. Additionally, these patients can bring objective data to their health care provider about changes in their hand, allowing clinicians to make individualized and more precise treatment decisions while optimizing time spent in the patient-clinician visit.

**Figure 2:**
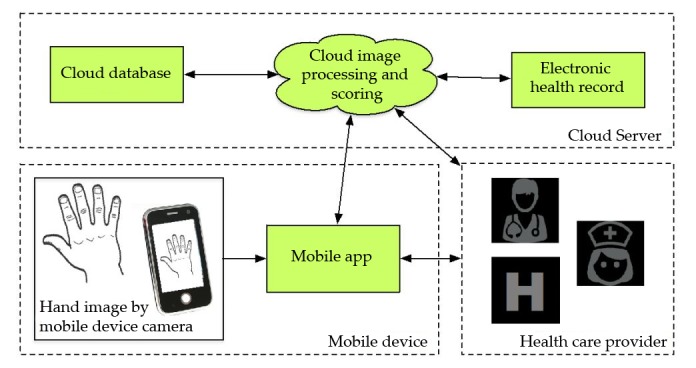
App system architecture.

**Figure 3:**
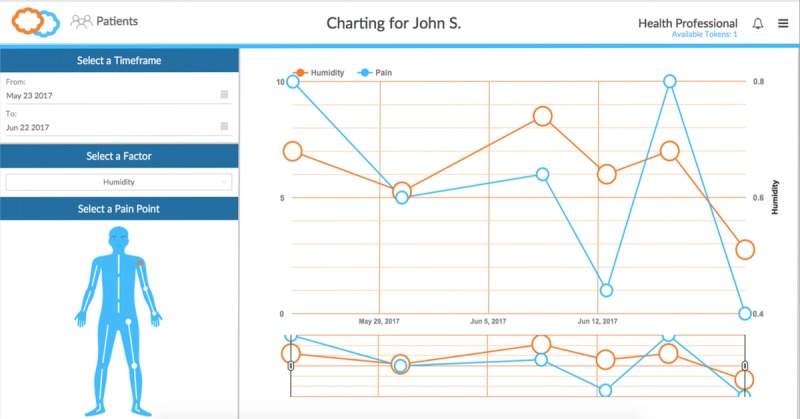
Web portal.

The LiveWith system architecture (see Figure 2) consists of (1) the mobile app that acts as an interface for the user, facilitates image capture, creates patient profiles and displays analytical results and (2) the Health Insurance Portability and Accountability Act (HIPPA)-compliant cloud server that stores patient profiles, manages data, and performs image processing. The Web portal (see Figure 3) provides a more advanced review of images and data trends and can be shared with clinicians or even integrated into an electronic health record.

**Figure 4:**
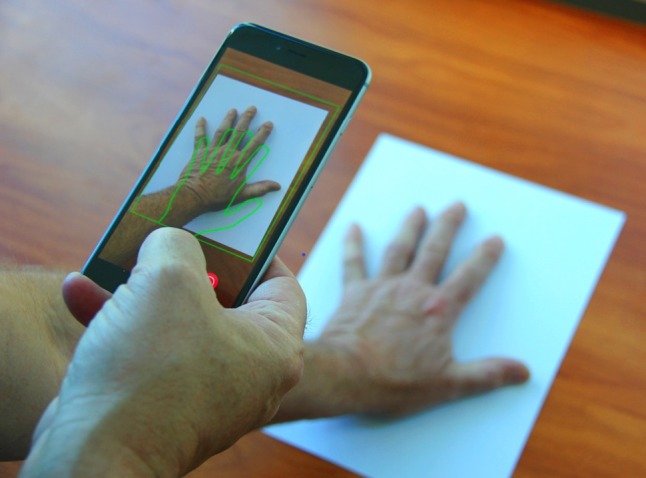
Hand optical imaging using letter size paper for calibration.

**Figure 5:**
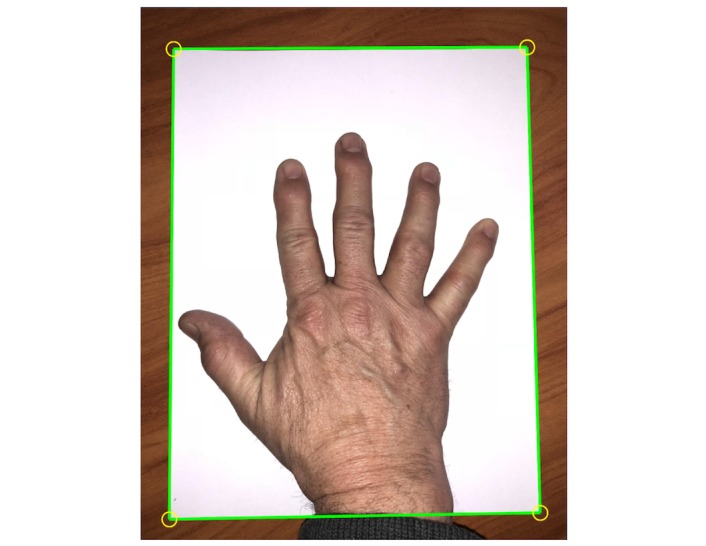
Imaging hand calibration requirements include (1) all 4 corners of the paper are in the photo, (2) the hand is within the paper's edges, (3) fingers are spread, and (4) the background behind the paper is darker and free of clutter.

**Figure 6:**
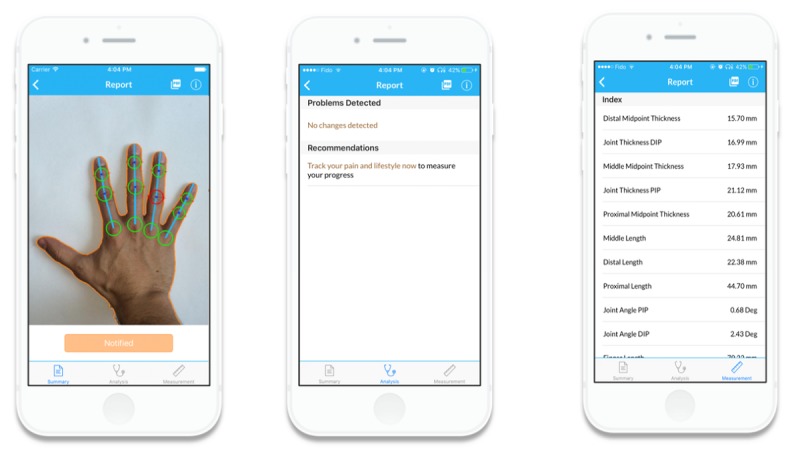
Output report including analyzed hand image, changes since last image, recommendations, and measurements of anatomical features of hand.

Hand analysis takes place after the patient takes a digital image of their hand with a standard sheet of white paper (letter size) in the background (see Figure 4). The sheet of paper, placed beneath the hand, provides a spatial calibration reference for the hand image. The measured dimensions of the paper boundary are paired with known dimensions of letter size paper for defining the spatial transformation of the image to real-world coordinates. Figure 5 shows the paper edges and corners detected by Hough transform. After this detection, the distances between the pixels now correspond to specific measurement units (ie, 100 pixel distance = 1.27 mm). The next steps are the segmentation of the hand from the white paper background of the now calibrated image and the detection of the hand boundary. The boundary pixel data are used to identify key anatomical features of the hand including fingertips and vertices and to measure hand geometry including finger and joint thickness, finger segment angulation, and interphalangeal joint angular deviation (see Figure 6).

### Design and Sample

This was a mixed-methods design. The quantitative portion was a traditional 2-group experimental design, and the qualitative portion was a follow-up telephone interview for intervention participants who did not complete the study. Participants were adults (aged over 18 years) with RA enrolled in a longitudinal cohort called the Rheumatology and Arthritis Investigational Network Database (RAIN-DB) [17,18]. To be included, participants had to be actively seeing a rheumatology provider at the researchers’ university rheumatology clinic, own a smartphone, and be able to read and speak English.

We enrolled 34 participants in the intervention group and 29 subjects in the control group. Data were collected at 2 visits, baseline and follow-up, which were 6 months apart. The baseline visit for intervention participants included meeting with a research assistant to download the app on the participant’s smartphone and to take an image of the hand to teach the patient how to use this feature of the app. Intervention participants were instructed to use the imaging technology at least once per month and the app for self-management regularly. Intervention participants were compensated US $50 for their time spent using the app. Control participants received usual care.

### Instruments

To evaluate whether the mobile app technology could improve self-management and self-efficacy, we used 2 questionnaires, the Patient-Reported Outcomes Measurement Information System (PROMIS) Self-Efficacy Managing Symptoms (P-SEMS) and the Patient Activation Measure (PAM).

#### Patient-Reported Outcomes Measurement Information System Self Efficacy in Managing Symptoms

The P-SEMS tool measures an individual’s perceived self-efficacy in managing the symptoms of their disease. Regarding health, self-efficacy is the foundation for an individual’s decision to act and implement healthy behaviors to manage their illness or symptoms and take control of their health through implementation of specific health behaviors to achieve improved outcomes [15,19]. Questions on the 28-item P-SEMS questionnaire assess the level of confidence a patient has in managing their symptoms in a variety of settings and situations such as, “I can keep my symptoms from interfering with the work I need to do” [19].

#### Patient Activation Measure

The PAM is a 13-item self-report instrument designed to assess an individual’s level of patient activation. It measures the patient’s self-reported knowledge, skills, and confidence in self-management of their chronic condition [16]. It asks the patient to either agree or disagree with statements such as, “When all is said and done, I am the person who is responsible for managing my health.” A highly activated individual is an involved and confident self-manager of their health. In those with chronic conditions, higher activation is associated with treatment adherence, improved self-monitoring, and appropriate care seeking [20]. Every 1-point increase in PAM score has been shown to correlate with a 2% decrease in hospitalization and 2% increase in medication adherence [21]. The PAM is a valid and reliable tool and has been tested in individuals with a variety of health conditions, in multiple languages, and across a variety of races and ethnicities [22,23].

#### Health Outcomes

Pain was measured using the Visual Analog Scale (VAS). The VAS measures pain from 0 to 10, with 0 meaning no pain and 10 being *worst pain* [24]. Physical function was measured with the reliable and valid 10-item questionnaire HAQ-II. Scores on the HAQ-II range from 0 (minimum loss of function) to 3 (completely disabled) [25].

#### Qualitative Interview

Intervention participants who did not complete the study were invited to participate in a qualitative telephone interview about the barriers to using the mobile app. Participants were asked, “tell me more about your experience using the app” with follow-up probes about their likes and dislikes of the app features, overall experience, and barriers to using the app. The qualitative interviews were transcribed verbatim.

### Analysis

Statistical analyses were conducted using Stata Statistical Software 15 (StataCorp, College Station, TX) [26]. We used *t* tests for comparison between groups on the PAM, P-SEMS, Pain, and HAQ-II. Pearson correlations were used to determine linear relationships between variables. Qualitative data were analyzed using content analysis.

## Results

### Sample Characteristics

Our final sample consisted of 21 intervention participants (62% original sample, 21/34) and 15 control participants (52% of original sample, 15/29). Intervention participants who withdrew from the study tended to have a slightly higher HAQ-II (0.63 vs 0.54) and pain score (3.2 vs 2.5) and lower PAM (67.4 vs 71.9) and P-SEMS (45.4 vs 47.3) at baseline compared with intervention participants who completed the study. These demographic changes were similar to the control group, with dropouts more likely to have a lower PAM (64.4 vs 68.8) and P-SEMS (46.4 vs 47.1) than controls who completed the study. Control participants who dropped out were more likely to have lower pain (2.6 vs 3.17) and lower HAQ-II (0.54 vs 0.63). None of these differences were statistically significant.

Our qualitative sample consisted of subjects who dropped out of the intervention group. We were able to reach and invite all 13 intervention participants who dropped out of the study. However, 1 intervention participant dropped out due to a diagnosis of lymphoma and ongoing health concerns, preventing her participation. The remaining 12 intervention participants who did not complete the study participated in the qualitative component of our study.

### Outcome Variables

The outcome variables are presented in Table 1.

**Table 1:** Quantitative outcome variables.

Variable	Intervention	Control	*P* value
P-SEMS^a^	2.80	−1.66	.04
PAM^b^	6.37	2.30	.46
HAQ-II^c^	0.02	0.05	.83
Pain	−0.61	0.18	.38

^a^P-SEMS: Patient-Reported Outcomes Measurement Information System Self-Efficacy Managing Symptoms.

^b^PAM: Patient Activation Measure.

^c^HAQ-II: Health Assessment Questionnaire-II.

#### Self-Efficacy in Managing Symptoms and Patient Activation

A 2-sample *t* test with equal variances for the P-SEMS showed that the intervention group had statistically significant improvement to their score compared with the control group (2.8 vs −1.66, *P*=.04). A 2-sample *t* test with equal variances showed that the intervention group had an increase in PAM score that was 2.8 times greater than the control group (6.37 vs 2.30, *P*=.48).

#### Health Assessment Questionnaire-II and Pain

Participants with higher PAM and P-SEMS scores had lower HAQ-II scores and lower pain scores at both baseline and follow-up. Changes in PAM and P-SEMS scores were negatively correlated with changes in HAQ-II (Pearson correlations: −0.33, *P*=.10 for PAM and −0.50, *P*=.007 for P-SEMS).

#### Qualitative Themes

There were 3 themes that arose from the qualitative interviews about the barriers to using the app for intervention participants who dropped out of the study. These themes were (1) frustration with technology, (2) RA made the app difficult to use, and (3) satisfaction with current self-management system. These themes and representative quotes are displayed in Table 2.

##### Frustration With Technology

Participants described frustration with technology especially with initial use of the app. Participants who did not have a good initial experience did not feel confident to return to the app or use it as directed on their own.

##### Rheumatoid Arthritis Made the App Difficult to Use

Participants who had more severe hand RA and who were disabled by their disease had difficulty using both the optical imaging and other features of the app. Some participants recruited someone to assist them with photographing their hand, but this also made it difficult for the participants to use the app on a regular basis.

##### Satisfaction With Current Self-Management System

Some participants explained that the mobile app could not be integrated easily into their lifestyle as they had already had a system to manage their RA. These participants felt that the use of the mobile app would not add to their self-management needs and that their system was working for them.

**Table 2:** Qualitative themes and representative quotes.

Theme	Representative quote
Frustration with technology	*Once they did get all the kinks out, it still wasn’t working for me to mess with it; so then, by that time, I had just never gone back in to do anything else with it.* [P2] *I dropped out a long time ago because of the problems they were having with the app at the very beginning, and I just got frustrated and I went, “Forget it.”* [P7]
Rheumatoid arthritis made the app difficult to use	*Because I live alone, and I have had my neighbors help me, and I just could not hold the telephone the right way, so the app really did not work for me.* [P5] *I could never get it to come out right, and I gave up. I just completely gave up. I could not get my hand photographed right, and there wasn’t always somebody there to help me with it.* [P11]
Satisfaction with current self-management system	*It’s so hit and miss, but, you know, I do my own logs as far as what I eat, what activities I do; and then, the days that I flare, I always go back and look and see if I did anything different, or ate anything different, to see if it coincides.* [P2] *My rheumatoid arthritis is under good control, so I don’t have a lot of flare-ups and that kind of thing; and if I go through a period where I do, to me, it’s most helpful just to write it in a journal.* [P1]

## Discussion

### Principal Findings

Our study showed a statistically significant improvement in P-SEMS and showed promising trends for improvement in PAM, HAQ-II, and Pain scores for participants who used the LiveWith app. These results demonstrated the potential of the LiveWith app for improving self-management and increasing confidence in these behaviors. Future studies with a larger sample size are necessary to confirm these findings.

Pilot studies are generally underpowered to assess evidence of benefit at the 95% CI; therefore, we find it especially promising that those participants who completed the intervention had statistically significant improvement in their self-efficacy scores [27,28]. As self-efficacy in managing symptoms increases, symptoms are more likely to be alleviated [20]. For example, pain has been shown to be inversely related to self-efficacy. By building self-efficacy in managing one’s own pain, the individual feels confident to use strategies to reduce pain intensity [14,15]. RA is truly an individual experience with different flares for each affected person. Empowering an individual with tools that can increase self-efficacy can improve symptoms and reduce the burden of this disease.

Our attrition rate was higher than the acceptable level of 20%, with a large percentage of both intervention and control group participants dropping out [29]. On the basis of our qualitative interviews with participants who dropped out, we attribute attrition of the intervention group almost primarily to technology issues. We had technology difficulties at the beginning of the study requiring several updates, and the time frame required to work out the *bugs* tarnished the initial app experience for many. Although we believed our app was study-ready, future studies should consider additional pilot testing and personnel training, or the use of a longer-standing technology platform. It seems critical that participant (or patient’s) first experience using the app is positive to promote confidence in continual use. Control group attrition may have been due to a lack of incentive and the already extensive paperwork required to participate in the longitudinal RAIN-DB. In addition, choosing a sample that was associated with a larger database and an actual physical appointment at the rheumatology clinic limited ongoing participation. Future studies should consider a completely digital experience with Web-based surveys and digital prompts to use the app and complete the tasks.

Although the purpose of our study was to learn more about self-managing behaviors, some participants believed collecting optical imaging hand data was the purpose of the study. Therefore, when there was difficulty using the optical imaging technology, the participants did not use the other self-managing aspects of the app. To our knowledge, there were no technology issues with the nonoptical imaging aspects of the app software, even as we went through various updates to the iOS and Android operating systems. Although the optical imaging is novel from a research and clinical perspective, it is unclear if this feature is essential to improve self-management behaviors. We believed that having the participants take an objective measurement of their hand would increase engagement and confidence in self-management. It is possible that requiring a hand image only once a month was too long of a time frame and reduced participant engagement with the app. We chose this length of time to watch for discernable changes in the hand and reduce overall burden to the patient. However, more frequent hand measuring may have reminded the participant about the day-to-day management of their disease and to use the other features of the app more regularly.

Participants who already had significant disability to the hand had difficulty operating the app. Some participants recruited others to aid them in using the app but found this to be additional work for them. It is assumed that traditional methods of self-management such as a pen-and-paper system may also have been difficult for this population. Future mobile app interventions should optimize voice technology and passive measurements to improve accessibility for patients with more progressive RA disability.

Some participants had pre-established routines of self-managing their disease through methods such as pen-and-paper journaling and felt it was sufficient. It is unclear whether a mobile app adds anything for a patient who already has a working system and is successfully managing their disease. Future studies should compare the use of a mobile app with pen-and-paper methods to see if simply using any self-management intervention shows improvement or if the mobile app shows superior improvement.

### Limitations

Our study had a small sample size and a large dropout rate. We experienced technology problems that caused delays and reduced the confidence of many of our participants. Our participants were a part of a larger longitudinal study and currently seeing a rheumatologist with regularity; therefore, they might have been more compliant than the general population. We did not include participants who completed the study in our qualitative interview process, which may have impacted our findings. Participants were required to own a smartphone to be included in the study; therefore, they might have had a more advanced knowledge of technology than the general population.

### Conclusions

The use of a mobile app with optical imaging capabilities appears to improve self-efficacy in managing symptoms of RA and may improve other outcomes such as patient activation, pain, and disability. Future research should include a larger sample size and comparisons with other self-management interventions, such as traditional pen and paper, to help determine if the use of this app mediates the improvement to health outcomes.

## Abbreviations

HAQ-II: Health Assessment Questionnaire-II
PAM: Patient Activation Measure
PROMIS: Patient-Reported Outcomes Measurement Information System
P-SEMS: PROMIS Self-Efficacy in Managing Symptoms
RA: rheumatoid arthritis
RAIN-DB: Rheumatology and Arthritis Investigational Network Database
VAS: Visual Analog Scale
